# High variation across *E. coli* hybrid isolates identified in metabolism-related biological pathways co-expressed with virulent genes

**DOI:** 10.1080/19490976.2023.2228042

**Published:** 2023-07-07

**Authors:** Rahul Gomes, Ashleigh Denison Kroschel, Stephanie Day, Rick Jansen

**Affiliations:** a Department of Computer Science, University of Wisconsin-Eau Claire, Eau Claire, WI, USA; b Department of Earth, Environment, and Geospatial Sciences, North Dakota State University, Fargo, ND, USA; c Masonic Cancer Center, University of Minnesota-Twin Cities, Minneapolis, MN, USA

**Keywords:** *E. coli*, EPEC, ETEC, WGCNA, enrichment analysis, differential expression

## Abstract

Virulent genes present in *Escherichia coli (E. coli)* can cause significant human diseases. These enteropathogenic *E. coli* (EPEC) and enterotoxigenic *E. coli* (ETEC) isolates with virulent genes show different expression levels when grown under diverse laboratory conditions. In this research, we have performed differential gene expression analysis using publicly available RNA-seq data on three pathogenic *E. coli* hybrid isolates in an attempt to characterize the variation in gene interactions that are altered by the presence or absence of virulent factors within the genome. Almost 26.7% of the common genes across these strains were found to be differentially expressed. Out of the 88 differentially expressed genes with virulent factors identified from PATRIC, nine were common in all these strains. A combination of Weighted Gene Co-Expression Network Analysis and Gene Ontology Enrichment Analysis reveals significant differences in gene co-expression involving virulent genes common among the three investigated strains. The co-expression pattern is observed to be especially variable among biological pathways involving metabolism-related genes. This suggests a potential difference in resource allocation or energy generation across the three isolates based on genomic variation.

## Introduction

Enteropathogenic *E. coli* (EPEC) is a public health concern as infection can lead to severe cases of diarrhea and is especially lethal among young children in developing countries.^1–5^ The major components of EPEC pathogenesis include the type III secretion system (T3SS) encoded by the locus of enterocyte effacement (LEE) island cluster region. A second pathogenicity island that encodes EspC is inserted into the genome by insertion elements or phages and helps develop the bundle forming pilus (BFP).^6–11^ These elements are involved in translocation of bacterial factors into host cells and host cell adherence. In the past, EPEC pathogenesis and virulence factor (VF) regulation have primarily focused on only a few prototype isolates (E2348/69, B171, E22, and E110019).^12–14^ More recently, global transcriptional analysis of pathogens has identified genome-wide transcription that promotes pathogenesis or novel virulence-associated factors.^15–20^

Global gene expression of EHEC has been investigated under several conditions, such as (1) response to nutrient limitation, (2) exposure to host cells, and (3) during growth phases.^15,21–23^

ETEC is a significant contributor to bacterial diarrheal disease. ETEC creates two unique toxins (LT-heat labile toxin and ST-heat stable toxin) that cause diarrhea by inducing the intestines to discharge an excessive amount of fluid. Frequent, watery diarrhea, cramping in the abdomen, fever, and nausea are some of the signs of an ETEC infection. EPEC and some Shiga toxin-producing *E. coli* (STEC) are two types of *E. coli* that cause enteric disease. Both types of *E. coli* have a gene cluster LEE, which is found on a chromosomal pathogenicity island. EPEC frequently causes sporadic diarrhea in adults and frequent infantile diarrhea (outbreaks). It is, however, noninvasive and nontoxic. The chromosomal LEE gene is responsible for the production of attaching and effacing lesions, which damage the brush border epithelium and result in increased secretion and watery diarrhea.^10,24,25^ Each of the nine definite *E. coli* pathotypes, including EPEC and ETEC, has a specific set of virulence factors (some in common across several pathotypes) that lead to the observed clinical virulence and pathogenicity.^26^ A recent review summarizes our current understanding of the activity/function that different virulence factors have across the nine *E. coli* pathotypes.^27^

The regulation of EPEC virulence genes involves numerous transcriptional factors and can be affected by environmental conditions and cell density.^28–31,7,32–37^ Several genes have been identified as important regulators of VFs. The first gene in the LEE1 operon, LEE-encoded regulator (Ler), positively modulates the expression of other LEE operons through promoter activation.^38^ In addition to regulation by external factors such as host body temperature and growth conditions, Ler can auto-regulate thereby utilizing an internal balance mechanism to maximize colonization efficiency while limiting host immune detection.^39–41^ Global regulators of Ler operon, GrlA and GrlR, promote and repress the expression of virulence genes, respectively.^42^ The EPEC adherence factor (EAF) plasmid can contain a plasmid-encoded regulator (Per) operon that is transcribed as a single polycistronic mRNA and independent of GrlA can activate LEE1.^43–45,32^

In this study, our objective was to identify which genes across three EPEC/ETEC hybrid strains were correlated with differentially expressed virulent genes in an attempt to understand the impact of the presence of virulent factors on *E. coli* biological processes. Additionally, we wanted to determine the impact of media (i.e., environment) on the expression of these virulent genes and the correlation structure between virulence genes and other differentially expressed genes. The aim was to identify how different *E. coli* strains may alter resource allocation to different biological processes under different conditions. Such interaction may also allow the investigation of virulence gene expression in emerging virulent multidrug resistance (MDR) strains. MDR has increased all over the world which is considered a public health threat. Several recent investigations reported the emergence of virulent multidrug-resistant bacterial pathogens from different origins that increase the necessity for the proper use of antibiotics.^46–50^ Results from differential expression combined with a gene enrichment analysis allow us to hypothesize the potential role environmental nutrient availability may play in virulent gene expression, *E. coli* strain pathogenicity, and provide a gene list of potential treatment targets.

## Materials and methods

### RNA-seq data

**Figure 1.**
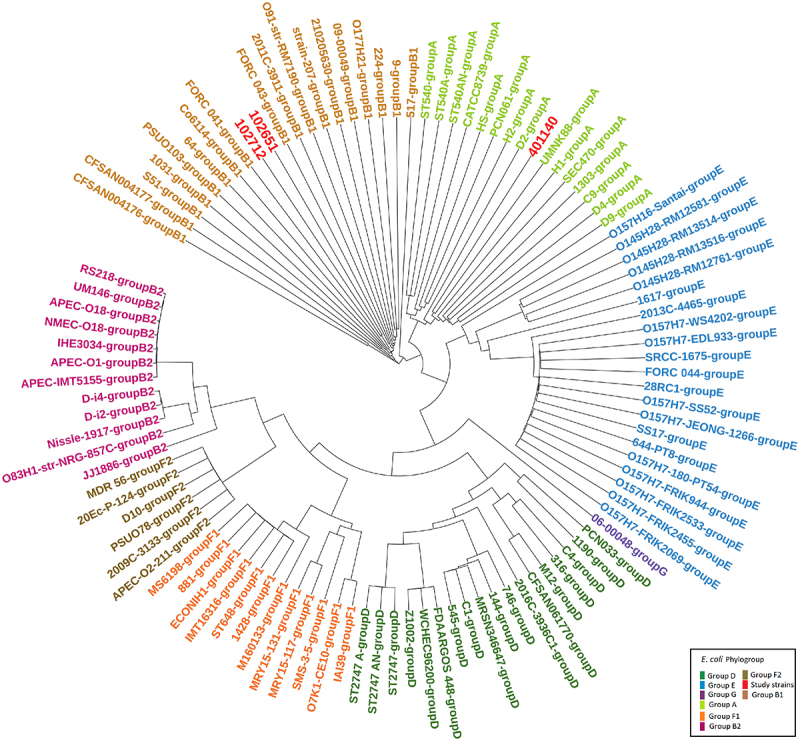
Phylogenetic tree depicting natural history of the *E. coli* genomes. 102651, 1026712, and 401140 are in red. Core genes were aligned using Multiple Sequence Alignment based on Fast Fourier transform (MAFFT)^52^ in the Rapid Large-Scale Prokaryote Pan Genome Analysis (Roary)^53^ pipeline. RAxML was used for tree generation using 1000 trees for bootstrapping, and bootstrap convergence assessment using autoMRE criterion stopped after 200 trees. Hybrid strains were evaluated by passing the output of RAxML through ClonalFrameML using a transition/transversion ratio of 2.5.

In the first stage of our research, 24 publicly available RNA-seq expressions were downloaded for three EPEC/ETEC hybrid isolates, namely 102651, 102712, and 401140 (Table A1). These hybrid isolates were reported by Hazen et al.^51^ The paper analyzed these three hybrid isolates. Since they contained the LEE region, these hybrid isolates possessed the features of EPEC. 102651 and 102712 also possessed the eltA and eltB genes responsible for heat-labile toxin which is common for many ETECs. 401140 contained the eatA gene which is also a feature of ETECs. The presence of features from both EPEC and ETEC makes them hybrid isolates. After performing global transcriptional analysis through RNA-sequencing, Hazen et al.^51^ identified these hybrid isolates to show variation in virulent gene content based on laboratory growth conditions. The authors also reported based on phylogenomic analysis that these hybrid isolates originated from EPEC and acquired ETEC virulent genes. Based on their research findings and the presence of both ETEC/EPEC virulent factors, these three strains were selected for our research. These strains also showed variations in their degree of evolutionary genomic differences and that relationship is visualized in Figure 1. Table 1 shows sample information and experimental conditions.

**Table 1.** RNA-seq sample information for the strains and experimental conditions selected for use in this study^51^

Lineage	Growth conditions				Replicates	Total samples
EPEC7	LB^1^	LBB^2^	DMEM^3^	DMEMB^4^	2	8
EPEC7	LB	LBB	DMEM	DMEMB	2	8
EPEC5	LB	LBB	DMEM	DMEMB	2	8

Abbreviations: ^1^Luria-Bertani medium, ^2^Luria-Bertani medium with added bile salts, ^3^Dulbecco’s Modified EagleMedium, ^4^Dulbecco’s Modified EagleMedium with added bile salts.

### Differential expression analysis

The first goal in analyzing these RNA-seq datasets is to identify differentially expressed genes across and within growth conditions. Mapping of the FASTQ files to the reference genomes available in the NCBI database was completed using Burrows-Wheeler Alignment Tool. This tool provides the BWA-MEM algorithm,^54^ minimizes sequencing errors, and is able to map sequences of around 100 bp with superior performance. After this step in the analysis, new alignment files are generated in Sequence Alignment Map (SAM) format which is converted to Binary Alignment Map (BAM) format to save on storage space. With the genomecov function in bedtools,^55^ we were able to compute the coverage of sequence alignments in BAM files using the reference genome. The total count data across multiple conditions per sample were combined, and genome annotation for each strain was completed using a General Feature Format (GFF) files generated by Prokka.^56^ Finally, these annotated files were used to identify common genes across all strains using the Roary^53^ pan genome pipeline. Processing was completed on North Dakota State University CCAST Servers.

**Figure A1.**
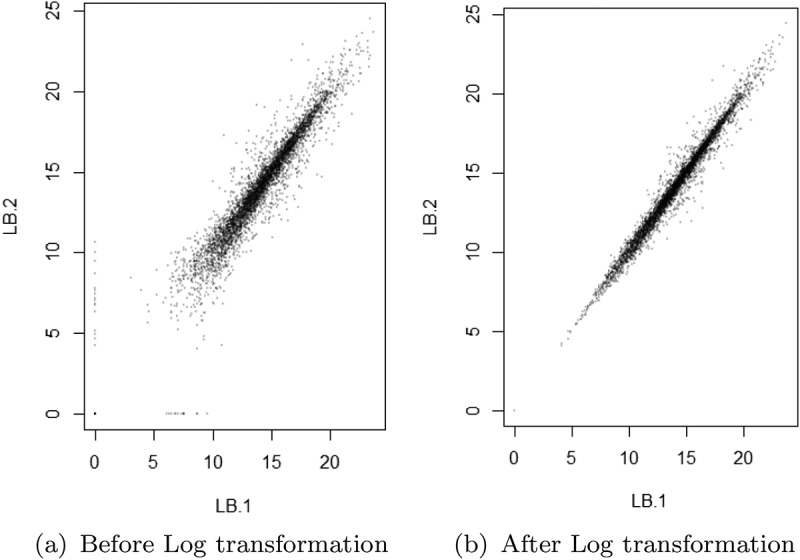
Rlog transformation was carried out before analysis: (a) Distribution of expression values in two LB mediums of 102651 before transformation. (b) Distribution after transformation.

Further analysis to obtain differentially expressed genes using these count data was completed using DESeq2.^57^ The process began by discarding reads from rRNA (all of them) since they distort the distribution of the other RNAs. An additional technical problem with estimating rRNA expression is that the mapping algorithm will mainly pile up the rRNA reads on the first copy in the genome and fail to distinguish expression from that first operon compared to the others (*E. coli* genomes range from 6 to 10 copies of rRNA operons). We used the regularized-logarithm (rlog) transformation offered by DESeq2 on the raw read counts. This transformation ensures that the genes with high counts do not affect the results extensively, while genes with lower counts have values attenuated toward genes’ averages. The rlog transformation uses ridge penalty to perform this operation ensuring that the generated read counts are approximately homoskedastic. The scatter plot in Figure A1 (see Appendix) shows the difference in values before and after transformation for isolate 102651. Then, differential expression (DE) of these genes was returned by the function for all three isolates. Any gene without expression data across all mediums was excluded in this study along with rRNA read counts. Differential expression was conducted as a pairwise comparison between two groups in every isolate. False Discovery Rate (FDR) values of <0.05 were considered to represent a significant differential expression for a gene.

### Co-expression network analysis

A co-expression network for each of these isolates was developed to further understand gene interactions using the Weighted Gene Co-expression Network Analysis (WGCNA) package.^58^ The first step toward WGCNA analysis is generating an undirected and weighted co-expression network where nodes are genes and edges represent the pairwise correlation between gene expressions. WGCNA offers the application of a soft-thresholding power, β, on the co-expression network to make it a scale-free topology.

As shown in Equation 1, through an iterative process, we raise the correlation of the genes $x_{i}$ and $x_{j}$ in the adjacency matrix to an extent that reduces noise in the dataset. For our experiments, we obtained β = 14, 14, and 12 for 102651, 102712, and 401140 respectively. This is followed by transforming the adjacency matrix into a Topological Overlap Matrix (TOM) which further reduces noise. The TOM output is then used to perform a hierarchical clustering of the genes using agglomerative clustering with average linkage. Highly co-expressed genes in branches are grouped together in a single cluster using a minimum set of genes per cluster. This is followed by merging groups/modules based on highly co-expressed profiles. Finally, Cytoscape^59^ is used for visualizing these network modules.

(1) $$a_{i}j=|cor(x_{i},x_{j}){|}^{\beta }$$

### Enrichment analysis

After the identification of differentially expressed genes and WGCNA modules, an enrichment analysis was performed separately on the modules that are associated with virulent genes. This technique reveals biological pathways that are more prevalent than would be predicted by random in a gene list.^60^ The tool used for this step was clusterProfiler^61^ which performed gene set enrichment analysis using the pathways reported in Gene Ontology (GO).^62^

## Results

The following section demonstrates the results obtained from analyzing the hybrid isolates and associated genes with virulent factors. Group variability in samples is done using PCA followed by differential gene expression analysis across four mediums. This is followed by the identification of genes with virulence factors followed by co-expression network analysis of these genes across the strains.

### Group variability in samples

**Figure 2.**
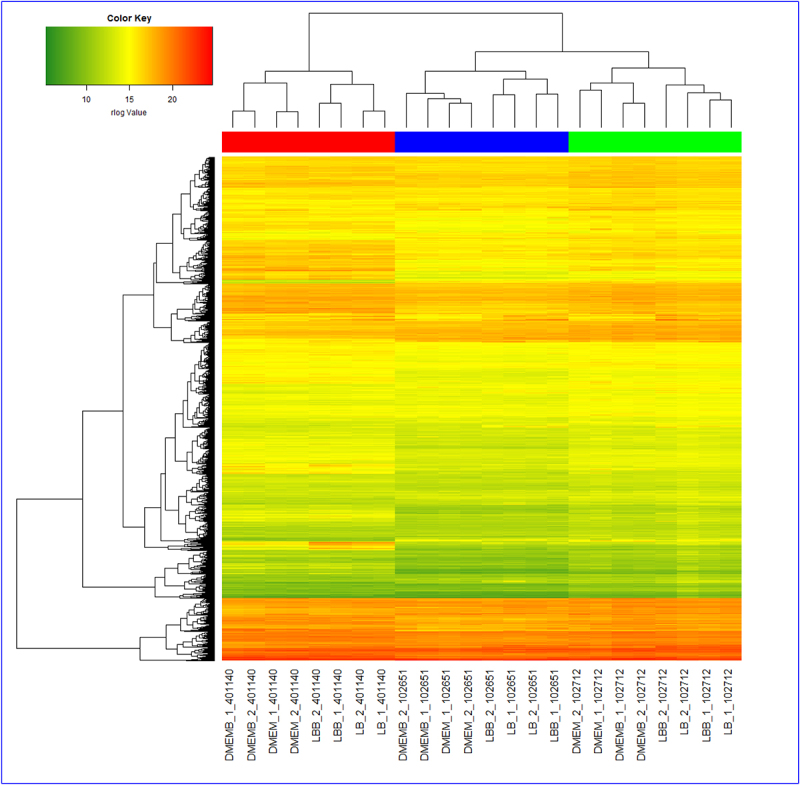
Heatmap showing hierarchical clustering of 3740 common genes (y-axis) in strains across all mediums. The x-axis labels take the format medium_sample_strain. The four medium types are DMEMB, DMEM, LBB, and LB, and the three *E.Coli* strains are 401140, 102651, and 102712. The colors in the heatmap represent rlog expression values with green having the lowest value and red the highest.

**Figure A2.**
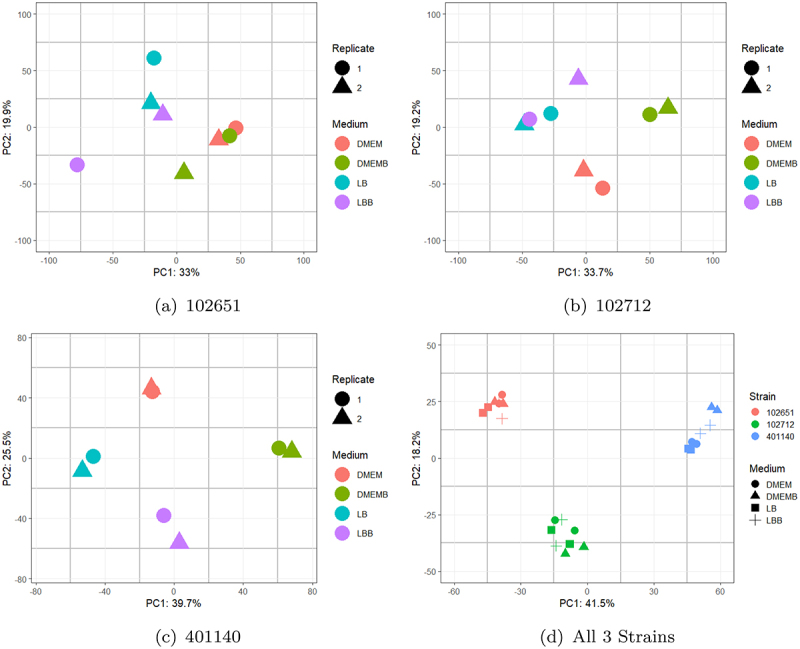
PCA for each isolate shown in (a), (b), and (c). Color represents medium and shape represents replicate number. All isolates have two replicates. Image (d) represents PCA for all isolates combined based on the sample growth medium.

After discarding rRNA reads and rlog transformation, principal component analysis (PCA) was applied which demonstrated that most of the replicates were grouped based on media type as shown in Figure A2 (see Appendix). In 102651 and 102712, the PCA was able to represent 52.9% of the variation, while 65.2% of the variation was demonstrated in 401140 using PC1 and PC2. A combined PCA of all three strains showed that 59.7% of variation could be explained using the first two principal components. The clustering of 3740 genes that were common across all strains is shown in Figure 2. The overall gene expression pattern here also demonstrates that the transcriptional pattern is heavily impacted by the growth medium. We also notice that the unique genomic content of each isolate plays a significant role in transcription.

### Differential expression analysis

Table 2 shows the number of differentially expressed genes across all three isolates. Differential expression analysis on the 3740 common genes in these hybrid *E. coli* isolates revealed 394 (10.5%) differentially expressed genes across DMEMB and DMEM and 510 (13.6%) across LB and DMEM medium. A total of 611 (16.3%) genes were differentially expressed while comparing isolates grown in LBB and DMEMB, while only 206 (5.5%) genes were observed to be differentially expressed for isolates grown in LBB and LB. In total, we observed that 998 of the 3740 (26.7%) common genes were differentially expressed in a minimum of one medium comparison.

**Table 2.** Differentially expressed genes across all mediums for the isolates 102651, 102712, and 401140. The first column shows which conditions were compared for the analysis. Results from the gray highlighted row were used for further analysis as it compared variation across all four mediums LB, LBB, DMEM, and DMEMB.

	102651			102712			401140		
	LFC1	LFC		LFC	LFC		LFC	LFC	
Conditions	>=2	<=-2	Total	>= 2	<= −2	Total	>=2	<=-2	Total
DMEMB^2^, DMEM^3^	19	18	37	50	154	204	305	113	418
LB^4^, DMEM	217	92	309	216	116	332	167	146	313
LBB^5^, LB	18	32	50	6	11	17	200	56	256
LBB, DMEMB	146	74	220	377	118	495	189	156	345
LB, LBB, DMEM, DMEMB	212	124	336	181	130	311	269	183	452

Abbreviations: ^1^Log-fold change, ^2^Dulbecco’s Modified Eagle Medium with added bile salts, ^3^Dulbecco’s Modified Eagle Medium, ^4^Luria-Bertani medium, ^5^Luria-Bertani medium with added bile salts.

### Virulence gene analysis

**Figure 3.**
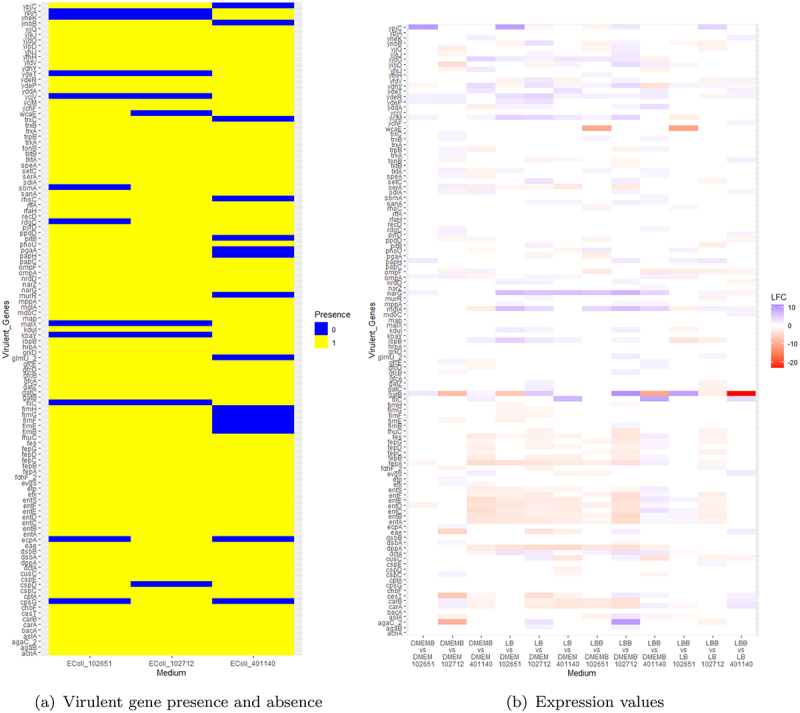
(a) Presence of virulent genes where yellow represents if they are present and blue if absent (b) their log-fold change (LFC) expression values across all three strains. The higher the intensity of the blue, the higher the log fold change (LFC) expression, and the higher the intensity of red, the more negative the LFC expression.

We explored how genes with virulent factors are distributed across these hybrid isolates. Information about specialty genes associated with these strains was derived from Pathosystems Resource Integration Center (PATRIC)^63^ database. After extracting specialty genes labeled as virulent factors, we were able to identify 115 virulent genes across these three isolates. Figure 3 highlights the distribution of virulent genes across these strains. After identifying the differentially expressed genes in these isolates, we filtered the dataset to obtain virulent genes that were differentially expressed again using FDR < 0.05. These data are shown in Figure 3 where differentially expressed virulent genes are either in blue or red. The higher the intensity of the blue, the higher the log fold change (LFC) gene expression, and the higher the intensity of red, the more negative the LFC gene expression.

**Figure 4.**
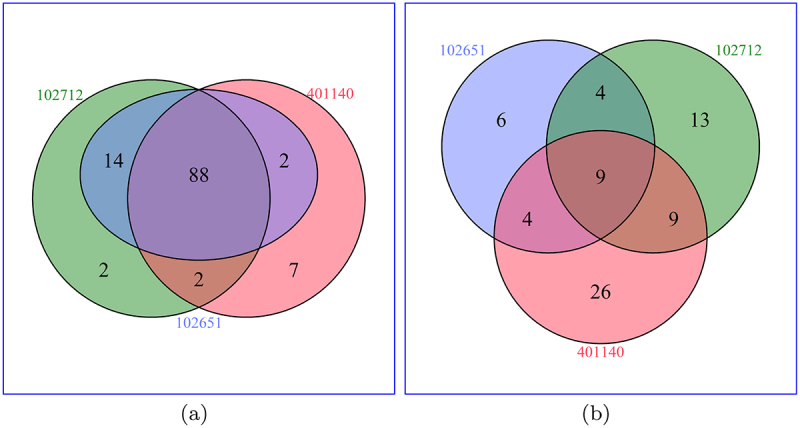
Venn diagram showing the number of virulent genes across strains before and after differential expression analysis. (a) Count of virulent genes appearing in each corresponding genome. (b) Count of significant differentially expressed virulent genes appearing in each corresponding genome.

The venn-diagram in Figure 4(a) displays the total number of virulent genes common in these strains, while the venn-diagram in Figure 4(b) shows the number of virulent genes that are differentially expressed. For the next stage of this research, we focused on how these differentially expressed virulent genes interact with other genes within each isolate. We see across all three strains that there are 88 virulent genes in common, but only nine (10.2%) of these genes are differentially expressed across all three strains.

Table 3 lists the nine differentially expressed genes with virulent factors found in all three isolates as identified in the previous section. We will focus our attention on how these virulent genes correlate with other differentially expressed genes in their respective strain genomes. To analyze this interaction, correlation networks were generated using Weighted Gene Co-Expression Network Analysis (WGCNA). A detailed explanation of the pipeline is provided in the next section.

**Table 3.** Log fold change (LFC) expression values for the nine virulent genes present in all three isolates 102651, 102712, and 401140 and differentially expressed across the three strains based on the FDR value.

	Isolate 102651		Isolate 102712		Isolate 401140	
Gene	LFC^1^	FDR^2^	LFC	FDR	LFC	FDR
*dppA*	−3.9879	0.0001	−4.0804	0.0001	−4.3249	0.0001
entD	−3.3532	0.004	−3.8348	0.0001	−2.5879	0.0001
fepA	−3.1938	0.0001	−3.8898	0.0001	−2.4826	0.0001
mglA	4.4666	0.0001	5.6775	0.0001	2.1198	0.0002
*narG*	7.0673	0.0001	6.8973	0.0001	4.9356	0.0001
ompF	−1.8085	0.0001	−2.2807	0.0001	−1.2814	0.0001
ycjM	2.5576	0.0129	3.7957	0.0001	2.2161	0.0001
*ydeP*	2.4542	0.0001	2.1313	0.0001	−1.2367	0.0007
ydeR	3.4075	0.0001	3.0454	0.0091	2.3551	0.0042

Abbreviations: ^1^Log-Fold change, ^2^False-Discovery rate.

### Co-expression network analysis

**Figure 5.**
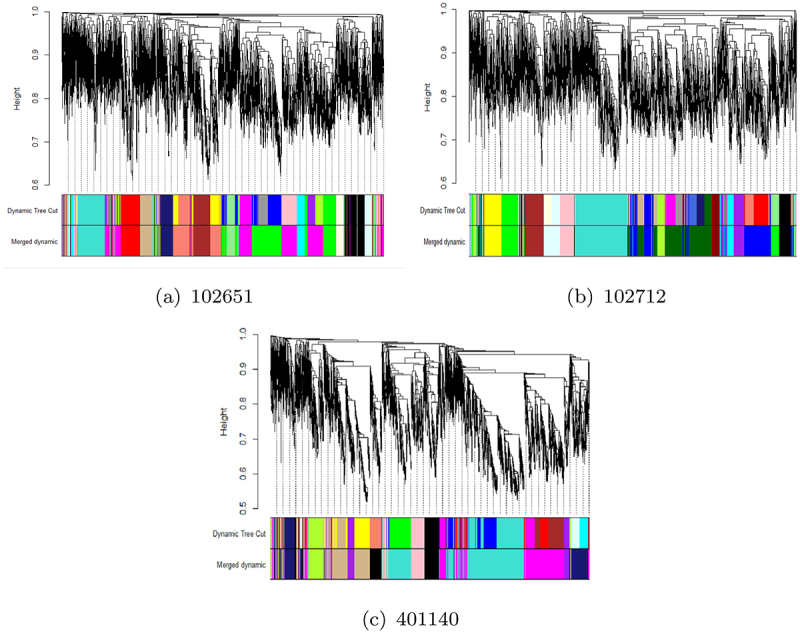
WGCNA results after clustering (top color band) and merging (bottom color band) groups for three isolates a: 102651, b: 102712, and c: 401140. These networks will be explored to study virulent gene interactions.

WGCNA analysis using soft-thresholding power was used to generate Topological Overlap Matrices (TOM) for all three strains. After analyzing the dendrograms in Figure 5, the minimum number of genes per module size was set to 100 to group genes with similar patterns and to facilitate network interaction. This process was able to identify 20 modules in 102651, 23 in 102712, and 17 for 401140. The dynamic tree-cut process was implemented. This approach merges modules that have a high degree of correlation in their genes. Modules with a degree of correlation of 0.75 or above were merged together into a single module. This step reduced the number of modules to 14, 14, and 9 in 102651, 102712, and 401140, respectively. The results of the clustering are shown in Figure 5

A summary of the modules is provided in Tables 4–6. It is observed that differentially expressed virulent genes are usually clustered in a single module showing a very high level of interaction. In strain 102651, there are four virulent genes in the brown module; in strain 102712, there are 14 differentially expressed virulent genes in the cyan module, while strain 401140 has nine virulent genes in the black module. In some situations, we also observed the virulent genes as hub genes (genes with the highest number of connections) for modules having a significant impact. This is the case for the black module in strain 102651 where the hub gene *ompA* is virulent, with strain 102712 for genes *cesT*, *entA*, and *entE* in brown and cyan modules. Based on the results in Table 4, we have four modules from strain 102651, five modules from strain 102712, and six modules from strain 401140 containing virulent genes highlighted in Table 3. These modules are highlighted in yellow in Tables 4–6. The common genes are in red. After extraction, strains 102651, 102712, and 401140 had a total of 248, 223, and 263 differentially expressed genes that were used to perform enrichment analysis. Together, the selected genes accounted for above 73%, 71%, and 58% of all the differentially expressed genes identified in the strains by DESeq2.

**Table 4.** Module hub genes in 102651 that were differentially expressed with functions and virulent genes identified by WGCNA. Yellow identifies the modules which contain virulent genes included in Table 3.

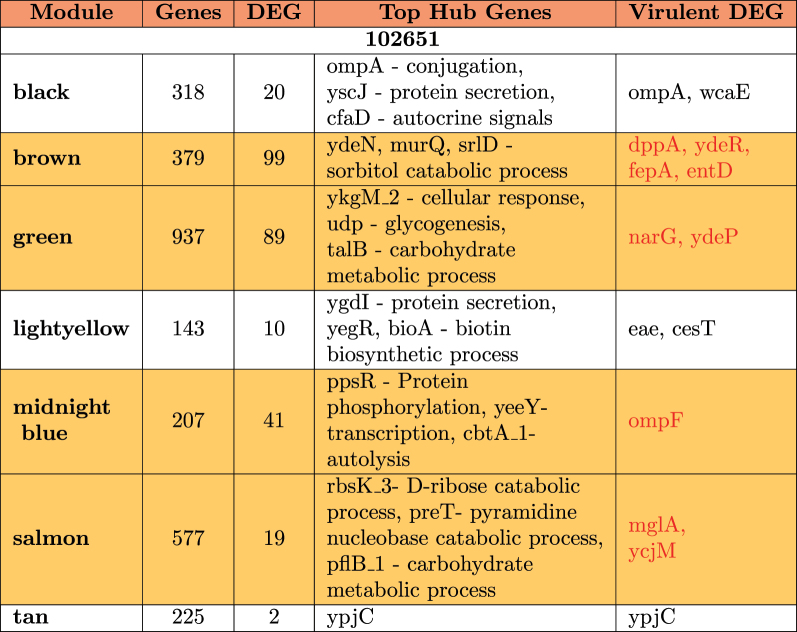

**Table 5.** Module hub genes in 102712 that were differentially expressed with functions and virulent genes identified by WGCNA. Yellow identifies the modules which contain virulent genes included in Table 3.

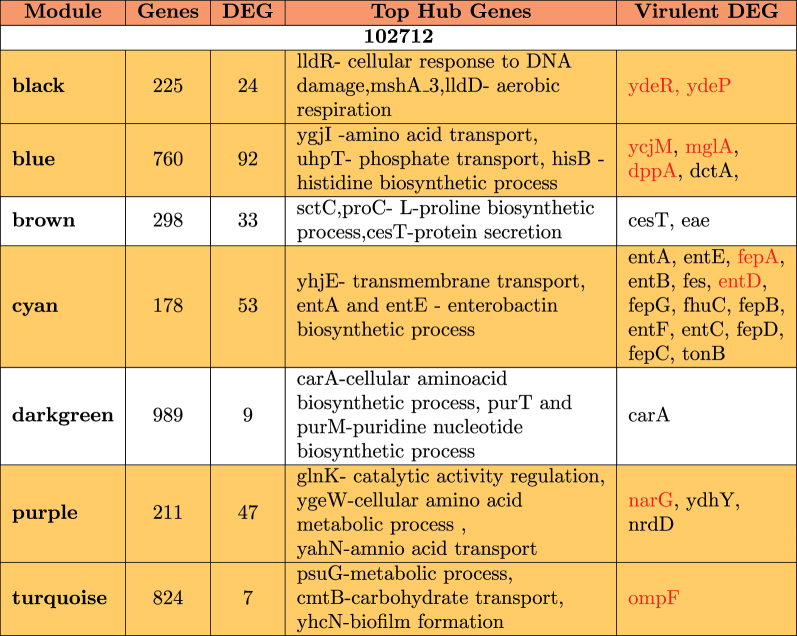

**Table 6.** Module hub genes in 401140 that were differentially expressed with functions and virulent genes identified by WGCNA. Yellow identifies the modules which contain virulent genes included in Table 3.

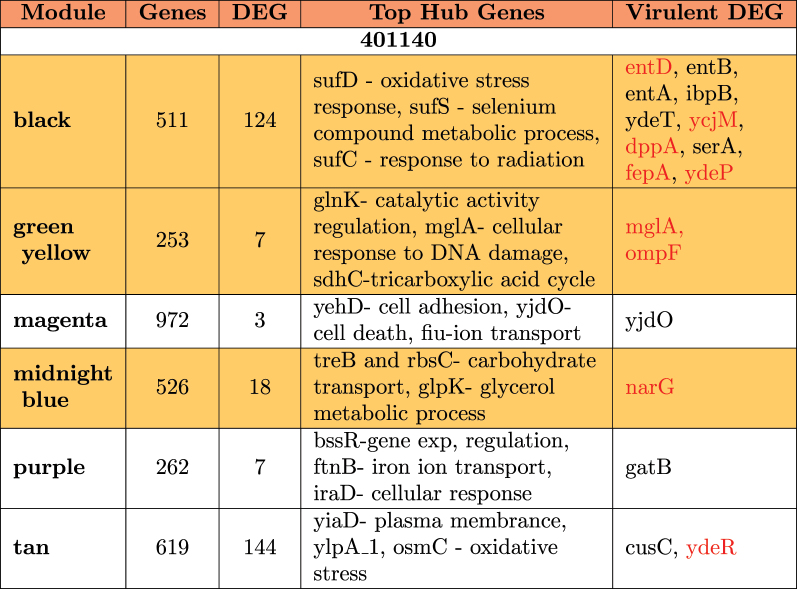

**Figure 6.**
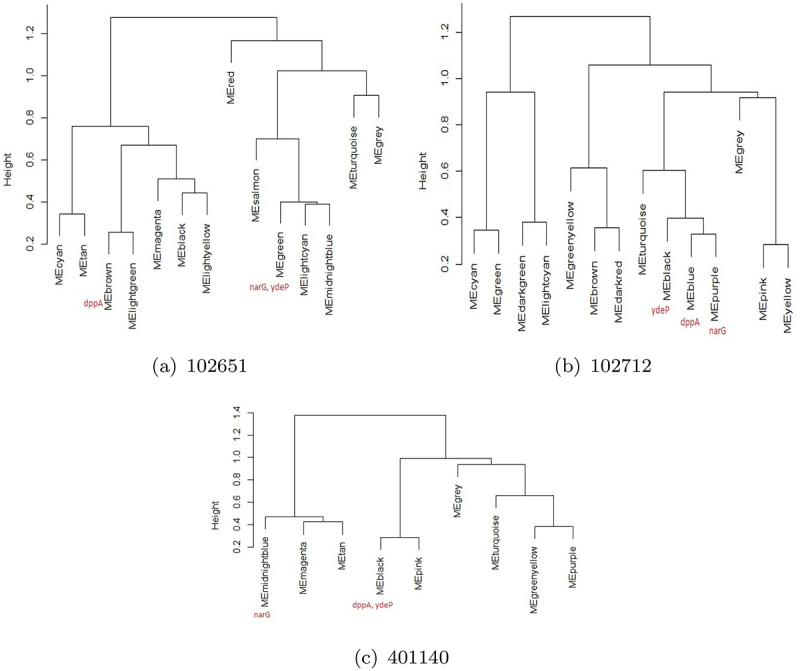
WGCNA hierarchical clustering of modules for three isolates (a) 102651, (b) 102712, and (c) 401140. The selected virulent genes (*dppA*, *narG*, *ydeP*) are identified in red in each tree. These virulent genes were differentially expressed.

**Figure 7.**
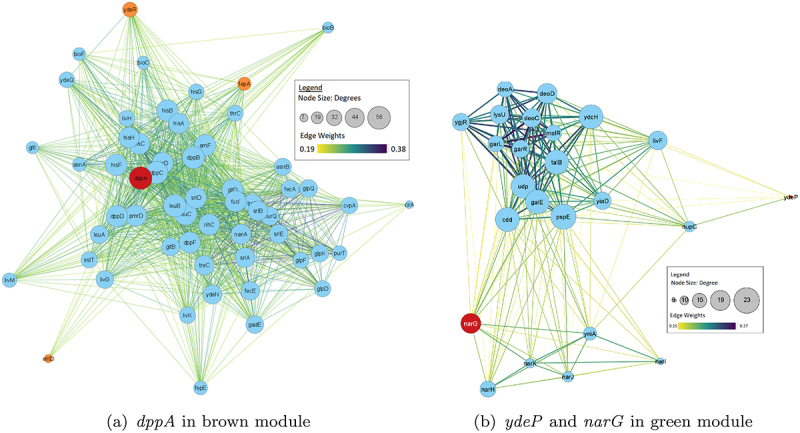
Network visualization of first neighbors using Cytoscape for strain 102651. Node size resembles its degree. It is observed that *dppA* is highly connected in the module. *ydeP* shows least connections. A larger node represents a higher number of connections. Larger edge weights are represented with thicker and blue lines.

**Figure 8.**
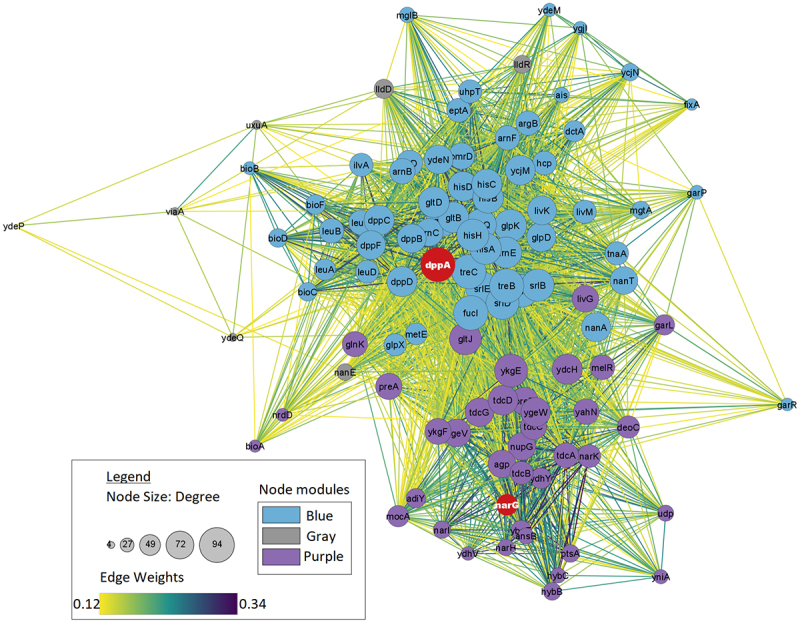
Network visualization of first neighbors for 102712 with the gray, blue, and purple module. Similar trend where *dppA* is highly connected in the module and *ydeP* shows the least connections. It is noticeable that *dppA* and *narG* have a significant interaction compared to 102651.

**Figure 9.**
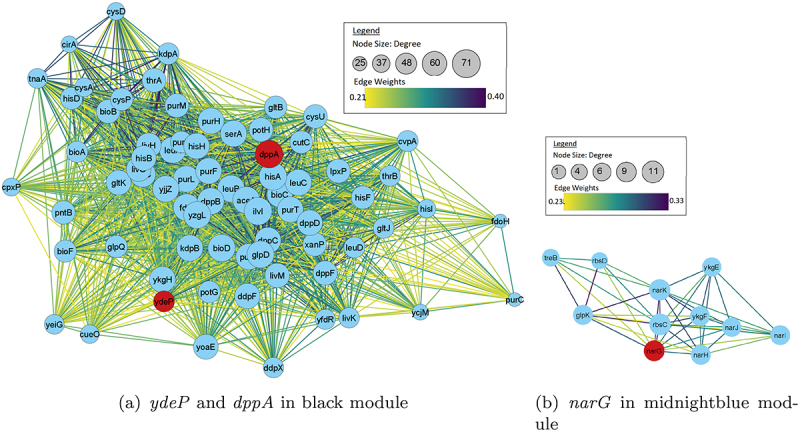
Network visualization of first neighbors using Cytoscape for strain 401140. Here *dppA* shows closer interaction with *ydeP*.

To further analyze strain differences, differentially expressed genes associated with *dppA*, *narG*, and *ydeP* were explored. These genes were selected based on their expression levels: showing the highest negative expression, highest positive expression, and significant difference in expression across all strains, respectively. Figure 6 shows how WGCNA modules are related to each other in these strains. It is observed that in strain 102651, *dppA* belongs to the brown module which is closely related to the light green module. Genes *narG* and *ydeP* are in green module which is significantly different from the brown module. In strain 102712, *dppA* was found in a blue module which is closely related to purple module and black module housing both *narG* and *ydeP*. Finally, in strain 401140, *dppA* and *ydeP* were both found in black module. However, the midnightblue module housing *narG* was significantly different in the clustering diagram. Hence, *narG* was analyzed separately. Figures 7–9 show the first neighbors based on the association between differentially expressed genes and *dppA*, *narG*, and *ydeP* derived using Cytoscape.^64^ The networks were filtered using the top 50th percentile of edge weights between these nodes.

### Enrichment analysis

**Figure 10.**
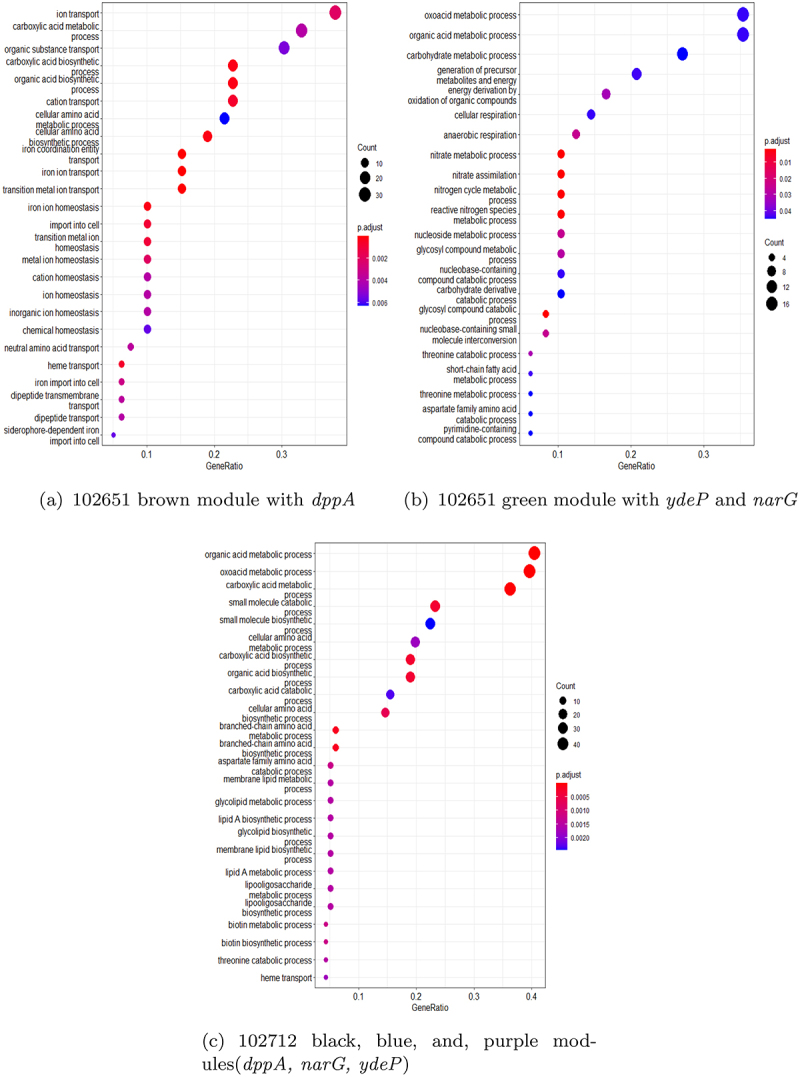
Biological pathways identified using genes from WGCNA modules with virulent genes for two isolates (a) 102651, and (b) 102712. The x-axis represents the gene ratio and the y-axis represents the identified biological pathways. The color and size of the plots represent the FDR and the count of genes in those biological pathways, respectively.

**Figure 11.**
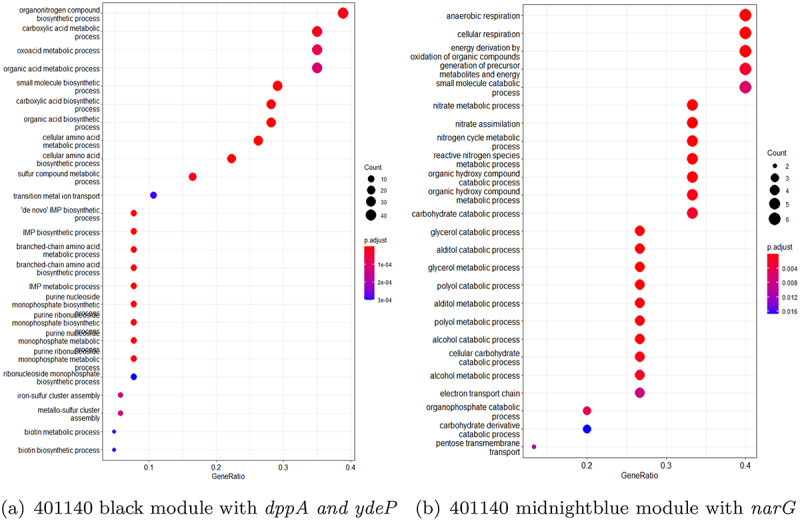
Biological pathways identified using genes from WGCNA modules with virulent genes for isolate 401140. The x-axis represents the gene ratio and the y-axis represents the identified biological pathways. The color and size of the plots represent the FDR and the count of genes in those biological pathways, respectively.

To identify the difference in the interaction of virulent genes across strains, we performed an enrichment analysis for differentially expressed genes associated with *dppA*, *narG*, and *ydeP*. Figures 10 and 11 show the results of the enrichment analysis in these three strains highlighting the top 25 biological processes associated with the three virulent genes. The analysis was performed using the clusterProfiler^61,65^ tool. This tool performed GO enrichment analysis^62^ by comparing genes identified in WGCNA modules with all the genes in the particular strain (referred as background genes). We compared these genes using the entire genome-wide annotation set for *E. coli* strain K12 available from org.EcK12.eg.db.^66^ The enrichment analysis uses hypergeometric testing to determine if a group of genes share similar function and are associated with any pathway. Prior to performing an enrichment analysis, genes in these strains were mapped to their corresponding Entrez IDs using clusterProfiler.^65^ There were only 22 biological pathways identified in green module of strain 102651 having *narG* and *ydeP*. In brown module of strain 102651 with gene *dppA*, 59 biological processes were identified. The GO categories were obtained using *p*values adjusted by Benjamini-Hochberg procedure with cutoff <0.05. For strain 102712, due to a high degree of correlation between black, blue, and purple modules, a GO enrichment analysis on the combined genes identified 52 biological processes. Finally, in strain 401140, the black module with genes *dppA* and *ydeP* was observed to have 84 biological processes, and genes in midnight blue module with *narG* were associated with 26 biological processes.

Enrichment analysis revealed interesting patterns in how the virulent genes were interacting with the other genes in the modules. Of the three virulence genes, only *dppA* and *NarG* formed unique interacting gene modules. The *dppA* gene in *E. coli* is a dipeptide-binding protein (DBP). Dipeptide-binding proteins inhibit heme binding. Some functions of this *dppA* gene include dipeptide/peptide transmembrane transporter activity and binding to heme and peptides.^67^ In Figure 7a, *dppA* is involved with amino acid uptake and metabolism genes. Many of them are co-transcribed. As these are growing in different media – though both with amino acids in abundance. A comparative analysis of the biological processes associated with the WGCNA module with *dppA* is shown in Table 7. We noticed a pattern where the biological processes are similar for strain 102651 and strain 401140 but not strain 102712.

**Table 7.** Biological processes associated with *dppA* identified by GO enrichment analysis.

Biological Processes	GO_ID	102651	102712	401140
Iron coordination entity transport	1901678	Y	-	Y
Iron ion transport	0006826	Y	-	Y
Transition metal ion transport	0000041	Y	-	Y
Heme transport	0015886	Y	Y	Y
Vation transport	0006812	Y	-	Y
Ion transport	0006811	Y	-	-
Dipeptide transmembrane transport	0035442	Y	Y	Y
Dipeptide transport	0042938	Y	Y	Y
Organic substance transport	0071702	Y	Y	-
Transmembrane transport	0055085	Y	-	-
Ion transmembrane transport	0034220	Y	-	Y
Oligopeptide transmembrane transport	0035672	Y	Y	Y
Anion transport	0006820	Y	Y	-
Anion transmembrane transport	0098656	Y	Y	-
Oligopeptide transport	0006857	Y	-	Y
Nitrogen compound transport	0071705	-	-	Y

Respiratory nitrate reductase 1 alpha chain (*narG*) is an enzyme complex that uses nitrate as an electron acceptor during anaerobic growth. *NarG* has many functions including, but not limited to, electron transfer activity, metal ion binding, nitrate assimilation, and nitrate metabolic process. *NarG* is also involved in GspD assembly and LF secretion by introducing nitrate into the system as shown in Table 8. Similarity was also observed here between 102651 and 401140.

**Table 8.** Biological processes associated with *narG* identified by GO enrichment analysis.

Biological Processes	GO_ID	102651	102712	401140
Nitrate metabolic process	0042126	Y	-	Y
Nitrate assimilation	0042128	Y	-	Y
Nitrogen cycle metabolic process	0071941	Y	-	Y
Reactive nitrogen species metabolic process	2001057	Y	Y	Y
Anaerobic respiration	0009061	Y	-	Y
Energy derivation by oxidation of organic compounds	0015980	Y	-	Y
Generation of precursor metabolites and energy	0006091	Y	-	Y
Oxoacid metabolic process	0043436	Y	Y	-
Cellular respiration	0045333	Y	-	Y
Organic acid metabolic process	0006082	Y	Y	-
Rlectron transport chain	0022900	-	-	Y

## Discussion

We observed that among the three EPEC/ETEC strains there were unique gene expression patterns. When looking at the 3,740 common genes, expression patterns varied across medium and across strain with most expression differences occurring for the comparison of LBB to DMEMB. Constructing gene expression networks demonstrated high clustering to the same module among virulence genes. Of the virulence genes, nine were common virulence genes identified to be differentially expressed in each of the three strains. Building gene networks for three of these virulence genes (*dppA*, *narG*, and *ydeP*) which demonstrated the largest differences showed both unique and common gene correlations with other genes across the *E. coli* genome.

The differences we observed in the co-expression of virulence factors and metabolism-related genes across the three *E. coli* isolates suggest that gene networks are dependent on genome content in different strains. This further suggests that potential clinical treatments may be less effective for specific isolates because of the differences in these gene interactions, but further research is needed.

Previous studies have provided evidence that resource/nutrient allocation is directed toward either the promotion of virulence factor or other cellular functions like growth depending on the environmental conditions.^9,68,69^ The regulators of virulence factors include both those that are pathogenic-specific and those that are common across *E. coli* strains.^7^ Our research suggests that there is variation in this process across *E. coli* strains, potentially leading to variations in the severity of disease within the host. Observations that the correlations between virulence factors and biological pathways varied by strain further support this hypothesis. Among the three virulence factors investigated in more detail here, *dppA* is uniquely highly correlated with pathways related to metal transporter genes while *narG* is uniquely highly correlated with pathways related to respiration and energy production.

Research byAlteri, Smith, & Mobley^70^ investigated the importance of bacterial metabolism while invading host cells and was able to identify the importance of mutant strains *dppA* and *oppA* as important to the fitness of uropathogenic *E. coli* (UPEC) as they are responsible for peptide transport. Xu et al.^71^ also noted the significance of *dppA* in creating more outer membrane proteins (OMPs) associated with cell adhesion. In our study investigating EPEC/ETEC gene expression, we also observed that *dppA* was highly correlated with many transport and homeostasis genes, metabolic, catabolic, and processing genes.

Lu, Fu, Xie, & Jin^72^ identified that *narG* plays a significant role in the proper functioning of terminal electron acceptors, which are crucial to the secretion of heat-labile enterotoxin (LT) in ETEC strains under anaerobic conditions. LT is a major virulence factor and can cause severe diarrhea. In strain 102651, *narG* identified in the green module was associated with 10 out of 22 biological processes. A similar pattern was observed in strain 401140 where it was associated with nine of the 26 biological processes in the midnight blue module. However, we did not observe significant interaction of *narG* in 102712, where it was only associated with three of the 52 biological processes identified in the black, blue, and purple modules.

Masuda & Church^73^ stated that *ydeP* is a novel gene related to acid resistance. It has many cofactor binding sites and responds to acidic pH levels. It works with cluster binding and formate dehydrogenase activity. This gene is a part of the prokaryotic molybdopterin containing oxidoreductase family. While *ydeP* showed significant variation in expression level, we noticed any unique biological processes that it was associated with the enrichment analysis. This may be due to its lower expression level across all the strains.

## Conclusion

In this research, we were able to identify how virulent genes show differences in expression levels across various strains even within the same growth medium. Differential expression analysis followed by the generation of WGCNA modules further demonstrates potential differential gene correlations with virulence factor expression across strains. Enrichment analysis is used to provide a potential biological impact that these virulent gene expression-level variations may have and provides a reduced list of future treatment targets in research. A limitation of the current study is that the publicly available dataset only included duplicate instead of triplicate data. This limits our ability to provide and demonstrate our results in a more robust way. Future work will explore these hybrid EPEC/ETEC isolates along with samples having more number of replicates. This will enable a robust differential expression analysis.^74^ Coupled with modern gene regulatory algorithms,^75^ we will explore molecular biology from these gene interactions in an optimized manner.

## Data Availability

This is a secondary data analysis. The original data that support the findings of this study are openly available at https://www.ncbi.nlm.nih.gov/. The accession numbers can be found in^51^ https://doi.org/10.1038/s41598-017-03489-z.

## Appendix A

**Table A1.** RNA seq. samples analyzed for comparative analysis.

Strain	SRA Accession	Medium-Replicate	Genome Accession	Phylogroup
102651	SRS566236	LB-1	GCA_002269745.1	B1
	SRS566243	LB-2		
	SRS566244	LBB-1		
	SRS566245	LBB-2		
	SRS566265	DMEM-1		
	SRS566266	DMEM-2		
	SRS566268	DMEMB-1		
	SRS566269	DMEMB-2		
102712	SRS566161	LB-1	GCA_002269735.1	
	SRS566162	LB-2		
	SRS566163	LBB-1		
	SRS566164	LBB-2		
	SRS566160	DMEM-1		
	SRS566213	DMEM-2		
	SRS566228	DMEMB-1		
	SRS566235	DMEMB-2		
401140	SRS566270	LB-1	GCA_001265465.1	A
	SRS566276	LB-2		
	SRS567938	LBB-1		
	SRS567940	LBB-2		
	SRS566296	DMEM-1		
	SRS566297	DMEM-2		
	SRS567927	DMEMB-1		
	SRS567937	DMEMB-2		
